# Gestational diabetes and risk of cardiovascular disease: a scoping review

**Published:** 2014-01-07

**Authors:** Cyril Archambault, Roxane Arel, Kristian B Filion

**Affiliations:** Cyril Archambault is a medical student at McGill University, Montreal, Quebec.; Roxane Arel, MD, is a staff physician in the Department of Family Medicine, St. Mary's Hospital Center, and a lecturer in the Department of Family Medicine, McGill University, Montreal, Quebec.; Kristian B. Filion, PhD, is an Investigator in the Centre for Clinical Epidemiology of the Lady Davis Institute of the Jewish General Hospital and an Assistant Professor of Medicine in the Division of Clinical Epidemiology, McGill University, Montreal, Quebec.

## Abstract

**Background:**

Gestational diabetes mellitus is associated with an increased risk of incident type 2 diabetes and has deleterious effects on other cardiovascular risk factors. However, the effect of gestational diabetes on the risk of cardiovascular disease remains unclear. We conducted a scoping review of the literature to examine the association between these 2 conditions.

**Methods:**

We systematically searched the PubMed and Embase databases for studies examining the association between gestational diabetes and cardiovascular disease. We restricted our search to studies involving humans that were published in English or French. Outcomes of interest included acute coronary syndromes, angina, arrhythmia, coronary artery disease, heart failure, myocardial infarction, stroke, and composite end points with these outcomes.

**Results:**

A total of 11 publications (3 cohort studies [1 published as an abstract], 2 cross-sectional studies, 1 case–control study [published as an abstract], 4 narrative reviews, and 1 editorial) met our inclusion criteria. The 2 cohort studies published as full manuscripts were conducted in overlapping populations. The included studies reported a range of adjusted relative risks for incident cardiovascular disease, from not significant to 1.85 (95% confidence interval [CI] 1.21 to 2.82). Adjustment for subsequent type 2 diabetes mellitus attenuated the effects but with wide 95% CIs that spanned unity (range 1.13 [95% CI 0.67 to 1.89] to 1.56 [95% CI 1.00 to 2.43]).

**Interpretation:**

Available data suggest that gestational diabetes is associated with an increased risk of cardiovascular disease. However, these data are limited, and evidence regarding this association independent of the increased risk due to subsequent type 2 diabetes and other risk factors for cardiovascular disease remains inconclusive.

Gestational diabetes mellitus occurs in about 7% of all pregnancies in North America.^1^ For the majority of women affected, blood glucose returns to prepregnancy levels during the postpartum period, but previous studies have established a strong association between gestational diabetes and increased risk of type 2 diabetes mellitus later in life.^2^,^3^ In addition, women with gestational diabetes are more likely to experience obesity, dyslipidemia, and hypertension in later life than are women who have not had gestational diabetes.^4^,^5^ However, the effect of gestational diabetes on the risk of cardiovascular disease remains unclear. It is also uncertain whether any association that does exist between gestational diabetes and cardiovascular disease is independent of the increased risk of type 2 diabetes mellitus. We therefore conducted a scoping review to examine the association between gestational diabetes and cardiovascular disease and the role of type 2 diabetes mellitus in any observed association.

## Methods

### Data sources

We systematically searched the PubMed and Embase databases from inception to 5 June 2012 for studies examining the association between gestational diabetes and adverse cardiovascular outcomes. Medical Subject Heading (MeSH) terms and keywords for gestational diabetes ("gestational diabetes," "pregnancy- induced diabetes") were combined with those for cardiovascular disease ("acute coronary syndrome," "cardiovascular disease," "cardiovascular pregnancy complications," "coronary artery disease," "heart attack," "myocardial infarction," "pregnancy complications," "stroke," and "unstable angina"); our search strategies are reported in detail in Appendices A and B. We also restricted our search to studies conducted in humans and published in English or French. Using the etiology search filters ("hedges") developed by the Health Information Research Unit at McMaster University, 6 we further restricted our search to observational studies by applying the filter that achieved the best balance of specificity and sensitivity. Finally, we hand-searched the references of primary studies and relevant reviews to identify additional studies not found in our electronic search.

We conducted this scoping review according to a prespecified protocol based on the methods described by Arksey and O'Malley.^7^ We are reporting the review according to the guidelines described in the Preferred Reporting Items for Systematic Reviews and Meta- Analyses (PRISMA) statement.^8^

### Study selection

We included primary studies that met the following criteria: (1) the study population was pregnant women stratified by the presence or absence of selfreported or clinically diagnosed gestational diabetes; (2) the study reported cardiovascular disease as an outcome (e.g., acute coronary syndrome, angina, arrhythmia, coronary artery disease, heart failure, myocardial infarction, stroke, or a composite of these end points) by gestational diabetes status; (3) the study was conducted in humans; (4) the study was published in English or French; and (5) the study design was observational, including cohort, case–control, crosssectional, and hybrid designs. To ensure that all relevant studies would be included, poster summaries and conference abstracts were eligible for inclusion, despite the less rigorous peer review that they undergo. We also included reviews and editorials that explicitly discussed the association between gestational diabetes and cardiovascular disease. We excluded animal studies and those conducted in women with a known history of cardiovascular disease.

### Data extraction

Two reviewers (including C.A.) conducted data extraction independently, with disagreements resolved by consensus or, when necessary, by a third reviewer (K.B.F.). For each study, the reviewers extracted information regarding study design and period, definition of gestational diabetes used, country where the study was conducted, demographic and clinical characteristics of the study population, prevalence of gestational diabetes, and type and incidence of cardiovascular events. The primary outcome of interest was cardiovascular disease, and we extracted the definition of cardiovascular disease used in each study. Additional events of interest included acute coronary syndromes, angina, arrhythmia, coronary artery disease, heart failure, myocardial infarction, and stroke. Outcomes were extracted as count data and crude effect measures (i.e., odds ratios [ORs] or hazard ratios [HRs]) with corresponding 95% confidence intervals (CIs). If necessary, crude effect measures and 95% CIs were calculated from reported count data. In addition, we extracted the results of multivariable analyses that adjusted for potential confounders, as well as those that adjusted for subsequent type 2 diabetes mellitus. When necessary, we contacted the authors of included studies to resolve ambiguities and obtain additional information. For reviews and editorials, we extracted the main themes discussed to delineate the current state of the literature.

## Results

### Search results

**Figure 1:**
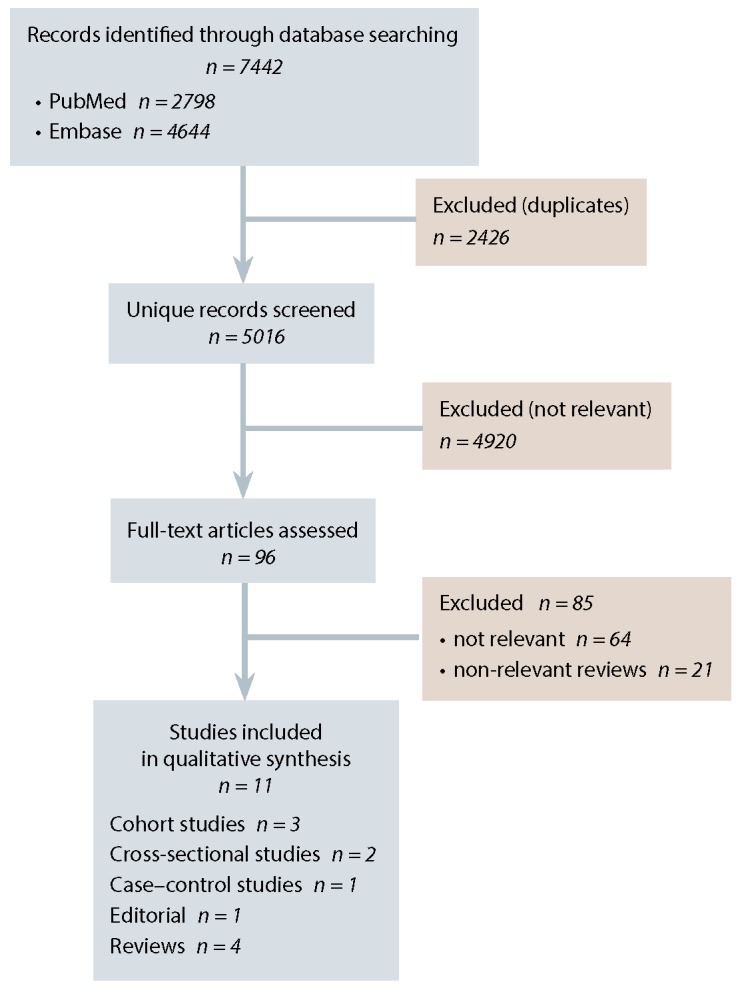
PRISMA flow diagram describing the systematic literature search for studies examining the association between gestational diabetes mellitus and cardiovascular disease.

Our initial search of PubMed and Embase identified 7442 potentially relevant publications (Figure 1). Following removal of 2426 duplicates, we excluded a further 4920 publications during screening of the title and abstract and examined the full texts of the remaining 96 articles. A total of 11 publications (4 full-length articles, 2 abstracts, 4 narrative reviews, and 1 editorial) met our inclusion criteria and were included in our scoping review. The 6 primary studies that met our inclusion criteria consisted of 2 crosssectional studies,^9^,^10^ 1 case–control study (published as an abstract),^11^ and 3 cohort studies (1 of which was published only as an abstract)^12^–^14^ (Table 1).

**Table 1:** Study and baseline patient characteristics of studies examining the association between gestational diabetes mellitus (GDM) and the risk of cardiovascular disease

Study	Definition of GDM	Country	Data source	Study period	No. (%) of patients		Age, yr, mean (SD)		Median follow-up, yr
					With GDM	Without GDM	With GDM	Without GDM	
**Cross-sectional**									
Carr et al.^9^ (n = 994)	Self-reported	USA	GENNID Study	1993–2001	332 (33.4)	662 (66.6)	48.6 (0.7)	52.4 (0.6)	NR
Freibert et al.^10^ (n = 3302)	Self-reported	USA	Kentucky Women's Health Registry	2006–2008	146 (4.4)	2 558 (77.5)*	57.1 (5.5)	60.3 (7.5)	NR
**Case–control**									
Schwarcz et al.^11^† (n = 27 443)	NR	Sweden	Swedish National Health Registry	1991–2008	NR‡	NR‡	NR‡		NR
**Cohort**									
Retnakaran and Shah^12^ (n = 435 696)	1 record of hospital admission or 2 ambulatory physician claims bearing diagnosis of diabetes or GDM between 120 days before and 180 days after delivery	Canada	Ontario administrative claims databases§	1994–1998	13 888 (3.2)	349 977 (80.3)||	31.1	29.2	12.5
Shah et al.^13^ (n = 89 453)	1 record of hospital admission or 2 ambulatory physician claims bearing diagnosis of diabetes or GDM between 120 days before and 180 days after delivery	Canada	Ontario administrative claims databases§	1994–1997	8 191 (9.2)	81 262 (90.8)	31¶	31¶	11.3
Bentley-Lewis et al.^14^† (n = 4010)	Carpenter–Coustan criteria	USA	Massachusetts General Hospital records	1998–2007	802 (20.0)	3 208 (80.0)	NR	NR	3.6

GENNID = Genetics of Non-Insulin dependent Diabetes, NR = not reported, SD = standard deviation.

* The non-GDM group was defined as patients with no history of preterm labour, pre-eclampsia, GDM, or third-trimester bleeding. The study by Freibert et al.10 included 3909 women but 607 of these women reported never having been pregnant. A total of 598 women (18.1% of the 3302 women who reported having been pregnant at least once) reported a history of these other pregnancy complications with no history of GDM; these women are not included in the n value for those without GDM shown in the table.

† Data extracted from poster summaries.

‡ This case–control study included 4653 cases (women with diagnosed cardiovascular disease) and 22 790 age-matched controls (women without diagnosed cardiovascular disease).

§ Administrative claims databases included population-based discharge abstract data, physician service claims, and demographic data. These data were linked to the Ontario Diabetes Database to exclude those with a pregestational history of diabetes.

|| The non-GDM group was defined as patients who did not undergo an antepartum glucose tolerance test. A total of 71 831 women (16.5% of the overall study group of 435 696) underwent an antepartum glucose tolerance test, suggesting the presence of an abnormal glucose challenge test result, but did not have GDM; these women are not included in the n value for those without GDM shown in the table.

¶ Mean age calculated for all participants (not subdivided by GDM status).

### Cross-sectional studies

In the 2 cross-sectional studies, Carr et al.^9^ and Freibert et al.^10^ used self-administered questionnaires to examine the association between a self-reported history of gestational diabetes and various cardiovascular disease end points. The study by Carr et al.,^9^ involving women with a family history of type 2 diabetes (n = 994), was part of the Genetics of Non-Insulin dependent Diabetes (GENNID) study. This multicentre US study was conducted between 1993 and 2001 and involved the collection of phenotypic data and genetic material.^9^ Freibert et al.^10^ conducted their study in women participating in the Kentucky Women's Health Registry (n = 3909). This registry collected data between 2006 and 2008 from a nonrandomized sample of women aged 18 to 89 years. Survey participants voluntarily completed detailed questionnaires about demographic characteristics and medical history, including both mental and physical health.^10^ Both of these cross-sectional studies involved US women of about 50 years of age^9^,^10^ and examined composite cardiovascular disease end points, as defined in Table 2. In both studies, the presence of gestational diabetes and cardiovascular disease was assessed only by self-administered questionnaire. The reference group in the study by Carr et al.^9^ consisted of women with no history of gestational diabetes, whereas the reference group used by Freibert et al.^10^ consisted of women with no history of gestational diabetes or other self-reported pregnancy complications, such as preterm labour, pre-eclampsia, or third-trimester bleeding.

**Table 2:** Effect of gestational diabetes mellitus (GDM) on the risk of cardiovascular disease

Study	Definition of cardiovascular disease	No. (%) of patients		Effect measure (95% CI)		
		With GDM	Without GDM	Crude	Adjusted	Adjusted for subsequent diabetes
**Cross-sectional**						
Carr et al.^9^	Self-reported history of coronary artery disease or stroke	51/329 (15.5)	81/653 (12.4)	OR 1.30 (0.89–1.89)	OR 1.85 (1.21–2.82)*	OR 1.56 (1.00–2.43)†
Freibert et al.^10^	Self-reported history of angina, heart attack, heart failure, or arrhythmia	64/146 (43.8)	573/2558 (22.4)‡	OR 2.70 (1.93–3.80)	NR	NR
**Case–control**						
Schwarcz et al.^11^§	ICD codes indicating presence of ischemic heart disease, stroke, or peripheral vascular disease	NA||	NA||	OR 1.98 (1.64–2.37)	OR 1.80 (1.49–2.18)¶	NR
**Cohort**						
Retnakaran and Shah^12^	Admission to hospital for acute myocardial infarction, coronary artery bypass, coronary angioplasty, stroke, or carotid endarterectomy	NR**	NR**	NR	HR 1.66 (1.30–2.13)††	HR 1.25 (0.96–1.62)
Shah et al.^13^	Admission to hospital for acute myocardial infarction, coronary artery bypass, coronary angioplasty, stroke, or carotid endarterectomy	NR	NR	HR 1.71 (1.08–2.69)	NR	HR 1.13 (0.67–1.89)‡‡
Bentley-Lewis et al.^14^ §	Cardiovascular disease as defined by ICD-9 codes	NR	NR	HR 1.32 (1.11–1.56)	NS§§	NR

CI = confidence interval, HR = hazard ratio, ICD = International Statistical Classification of Diseases and Related Health Problems, ICD-9 = ICD Ninth Revision, NA = not applicable, NR = not reported, NS = not significant, OR = odds ratio.

* OR adjusted for age, menopausal status, and clustering on the proband. Subsequent analyses adjusted for age, menopausal status, and race/ethnicity (OR 1.66, 95% CI 1.07–2.57). For this study, because of missing data, analyses were restricted to 329 women with GDM and 653 women without GDM.

† OR adjusted for type 2 diabetes and proband status.

‡ The non-GDM group was defined as patients with no history of preterm labour, pre-eclampsia, GDM, or third-trimester bleeding.

§ Data extracted from poster summaries.

|| The prevalence of a history of GDM was 3.5% among cases and 1.8% among controls.

¶ OR adjusted for potential confounders, specifically smoking, chronic hypertensive disease, and overweight (body mass index > 25 kg/m^2^).

** The rates of cardiovascular events were 4.2 per 10 000 person-years among women with GDM and 1.9 per 10 000 person-years among women who did not undergo an oral glucose tolerance test.

†† HR adjusted for age, year of delivery, rural residence, income, comorbidity, pre-existing hypertension, and gestational hypertension.

‡‡ Shah et al. did not adjust for potential confounders other than subsequent diabetes.

§§ HR was no longer significant after adjustment for age, systolic blood pressure, parity, and maternal weight gain during pregnancy; the exact HR was not reported.

In the study by Freibert et al.,^10^ the prevalence of cardiovascular disease was substantially greater among women with a history of gestational diabetes than among those with no history of gestational diabetes or other self-reported pregnancy complications (43.8% v. 22.4%; difference 21.4 percentage points, 95% CI 13.2 to 29.6 percentage points) (Table 2). In the study by Carr et al.,^9^ there was no difference in the crude prevalence of cardiovascular disease by gestational diabetes status (15.5% v. 12.4%; difference 3.1 percentage points, 95% CI –1.6 to 7.8 percentage points). However, gestational diabetes was associated with an increased prevalence of cardiovascular disease after adjustment for potential confounders, specifically age, menopausal status, and clustering on the proband (OR 1.85, 95% CI 1.21 to 2.82). Further adjustment for type 2 diabetes attenuated the association (OR 1.56, 95% CI 1.00 to 2.43).

In secondary analyses, Carr et al.^9^ stratified their results by race/ethnicity. Although there was some variability in the point estimate of the association between gestational diabetes and the prevalence of cardiovascular disease, all of the 95% CIs were wide and overlapping (for whites, OR 1.62, 95% CI 0.84 to 3.12; for African Americans, OR 1.27, 95% CI 0.62 to 2.61; for Latinas, OR 2.91, 95% CI 1.06 to 8.02).

We also examined individually the components of the composite cardiovascular end points in the 2 crosssectional studies^9^,^10^ (Table 3). After adjustment for potential confounders, gestational diabetes was associated with increased odds of angina, arrhythmia, coronary artery disease, and myocardial infarction. The analyses of associations with heart failure and stroke were inconclusive because of wide 95% CIs.

**Table 3:** Association between gestational diabetes mellitus (GDM) and individual cardiovascular end points

Event	Study*	No. (%) of patients		OR (95% CI)	
		With GDM	Without GDM	Crude	Adjusted
Angina	Freibert et al.^10^	17 (11.6)	113 (4.4)	2.85 (1.66–4.89)	2.90 (1.50–5.60)†
Arrhythmia	Freibert et al.^10^	41 (28.1)	386 (15.1)	2.20 (1.51–3.20)	2.40 (1.50–3.70)†
Coronary artery disease	Carr et al.^9^	40 (12.2)	70 (10.7)	1.29 (0.71–2.32)	1.58 (1.00–2.49)‡
Heart failure	Freibert et al.^10^	1 (0.7)	28 (1.1)	0.62 (0.08–4.61)	0.70 (0.10–5.60)†
Myocardial infarction	Freibert et al.^10^	5 (3.4)	46 (1.8)	1.94 (0.76–4.94)	3.40 (1.10–11.30)†
Stroke	Carr et al.^9^	19 (6.2)	31 (4.9)	1.15 (0.76–1.74)	1.67 (0.87–3.22)‡

CI = confidence interval, OR = odds ratio.

* Both studies examining individual end points were cross-sectional. In the study by Carr et al.^9^, the association between GDM and coronary artery disease was examined in 329 women with GDM and 653 women without GDM. The association between GDM and stroke was examined in 305 and 631 women, respectively.

† OR adjusted for age, education, and smoking status. The reference group for these analyses in the study by Freibert et al.^10^ was never-pregnant women. Compared with never-pregnant women, the adjusted ORs for women with a history of pregnancy but no history of GDM were 1.10 (95% CI 0.70–1.80) for angina, 1.10 (95% CI 0.90–1.50) for arrhythmia, 0.80 (95% CI 0.30–1.90) for heart failure, and 1.50 (95% CI 0.70–3.20) for myocardial infarction.

‡ OR adjusted for age, menopausal status, and clustering on the proband.

### Case–control study

We identified 1 case–control study examining the association between gestational diabetes and cardiovascular disease, published in 2011 as a conference abstract^11^ (Table 1). In that study, Schwarcz et al.^11^ used codes from the International Statistical Classification of Diseases and Related Health Problems (ICD) to identify women in the Swedish National Health Registry with diagnosed cardiovascular disease for the period 1991 to 2008 and selected agematched controls without cardiovascular disease, all with at least one birth during the study period. Women included as cases had ischemic heart disease, stroke, or peripheral vascular disease. The prevalence of a history of gestational diabetes was greater among cases than controls (3.5% v. 1.8%; OR 1.98, 95% CI 1.64 to 2.37) (Table 2). This association persisted after adjustment for potential confounders, specifically smoking, chronic hypertensive disease, and body mass index above 25 kg/m^2^ (HR 1.80, 95% CI 1.49 to 2.18).

### Cohort studies

We identified 3 cohort studies^12^–^14^ examining the association between gestational diabetes and cardiovascular disease, 1 of which was published only as an abstract^14^ (Table 1). The first 2 cohort studies were based on administrative data from the Canadian province of Ontario during overlapping study periods.^12^,^13^ Shah et al.^13^ examined the effect of gestational diabetes on the risk of incident cardiovascular disease between April 1994 and March 1997, whereas Retnakaran and Shah^12^ examined women with gestational diabetes between April 1994 and March 1998. In the former study,^13^ the primary exposure was gestational diabetes, whereas in the latter study,^12^ mild glucose intolerance was the primary exposure and gestational diabetes was a secondary exposure category. In both studies, gestational diabetes was defined as 1 hospital admission or 2 ambulatory physician claims bearing the diagnosis of diabetes or gestational diabetes between 120 days

before and 180 days after delivery.^12^,^13^ Patients with a previous diagnosis of nongestational diabetes (i.e., type 1 or type 2 diabetes) were excluded. Both studies also restricted inclusion to pregnancies that resulted in a live birth and randomly selected one pregnancy per woman. In both cohort studies, the median follow-up period was longer than 10 years, and the sample sizes were 89 453 women^13^ and 435 696 women,^12^ respectively. These 2 studies involved women of about 30 years of age.^12^,^13^ Shah et al.^13^ excluded women with a history of cardiovascular disease. Although Retnakaran and Shah^12^ did not apply this exclusion criterion, the proportion of women with a history of cardiovascular disease who were included in the study was exceedingly small (B. Shah, personal communication, 24 July 2012). In the third cohort study, published as an abstract in 2011, Bentley-Lewis et al.^14^ studied 802 women with gestational diabetes and 3208 women without the condition, matched on gravidity, who delivered at Massachusetts General Hospital between 1998 and 2007. Importantly, patients in whom type 2 diabetes developed after delivery were censored. In addition to examining the overall relationship between gestational diabetes and cardiovascular disease, Bentley-Lewis et al.^14^ conducted race-specific analyses to examine potential effect modification.

As with the cross-sectional and case–control studies, composite end points were used in the 2 Canadian cohort studies, and all 3 cohort studies used ICD codes to identify events (Table 2). For the 2 Canadian studies, the components of the composite end point were admission to hospital for acute myocardial infarction, coronary artery bypass, coronary angioplasty, stroke, or carotid endarterectomy.^12^,^13^ Bentley-Lewis et al.^14^ reported the use of ICD codes, but they did not report the components of their definition of cardiovascular disease or provide the corresponding codes. In the study by Retnakaran and Shah,^12^ women with gestational diabetes had a higher crude rate of cardiovascular disease than women without gestational diabetes (4.2 v. 1.9 per 10 000 person-years, respectively). After adjustment for potential confounders, gestational diabetes remained associated with a higher rate of incident cardiovascular disease (HR 1.66, 95% CI 1.30 to 2.13). However, further adjustment for subsequent type 2 diabetes attenuated this association and produced estimates that included both null and clinically important effects (HR 1.25, 95% CI 0.96 to 1.62). Similar results were obtained in the cohort study by Shah et al.^13^

In the study by Bentley-Lewis et al.,^14^ gestational diabetes was associated with an increased rate of cardiovascular disease (HR 1.32, 95% CI 1.11 to 1.56). However, after adjustment for age, systolic blood pressure, parity, and maternal weight gain during pregnancy, the association was no longer significant. Unfortunately, women in whom type 2 diabetes subsequently developed were censored, which makes the results difficult to interpret; such women have a greater risk of cardiovascular disease, and this nonrandom censoring may have resulted in selection bias. In a race-stratified analysis, Hispanic women with gestational diabetes were more likely to experience cardiovascular disease than Hispanic women without gestational diabetes (HR 1.70, 95% CI 1.60 to 2.41).^14^

### Reviews and editorials

The association between gestational diabetes and cardiovascular disease has been examined in 4 narrative reviews^15^–^18^ and 1 editorial.^19^ All of these publications referenced primary studies included in the present scoping review. The main themes discussed in these 5 papers are summarized in Table 4. Importantly, the consensus from these publications is that additional research is required to determine the effect of gestational diabetes on the risk of cardiovascular disease independent of traditional cardiovascular risk factors, and there remains a need to further examine potential effect modifiers of this relationship (e.g., race and age).

**Table 4:** Main themes discussed in narrative reviews and editorials of gestational diabetes mellitus and the risk of cardiovascular disease

Theme	References
GDM increases the risk of type 2 diabetes mellitus and metabolic syndrome occurring later in life.	15–18
"Hard" CVD events occur earlier in patients with GDM than in those without GDM.	17, 18
GDM may confer additional CVD risk beyond CVD risk factors, but the association is still unclear. Further research is required to determine the direct association of GDM and CVD.	15–19
GDM is associated with increased vascular inflammation and endothelial dysfunction.	15, 16, 18
Preliminary screening, medical therapies, and lifestyle changes may be important to reduce type 2 diabetes mellitus and CVD in women with previous GDM.	15–19

CVD = cardiovascular disease, GDM = gestational diabetes mellitus.

## Interpretation

Our study was designed to review the existing literature examining the association between gestational diabetes and cardiovascular disease and to examine the role of subsequent type 2 diabetes in this relationship. We identified 11 publications (6 primary research studies, 4 narrative reviews, and 1 editorial) discussing this association, and their data suggest that gestational diabetes is independently associated with an increase in the risk of cardiovascular disease. In the 2 large cohort studies based on Canadian administrative databases,^12^,^13^ further adjustment for subsequent diabetes attenuated the association and resulted in point estimates accompanied by imprecise 95% CIs that were unable to rule out either no association or clinically important increased risks. Thus, evidence regarding a potential increased risk of incident cardiovascular disease independent of the increased risk of subsequent type 2 diabetes and other cardiovascular risk factors remains inconclusive.

One of the key findings of this review is that highquality evidence regarding the effect of gestational diabetes on the risk of cardiovascular disease is limited. Only 6 English-language studies have examined this association to date: 2 cross-sectional studies that relied on self-reported exposure and outcome assessment,^9^,^10^ 2 underpowered cohort studies with overlapping study populations,^12^,^13^ and 2 studies published as conference abstracts only.^11^,^14^ The overlapping study populations of the 2 Canadian cohort studies likely artificially increased the homogeneity of the available data. Furthermore, all included studies were likely affected by residual confounding. For example, there was no adjustment for obesity, an important risk factor for both gestational diabetes^4^ and cardiovascular disease.^20^ Model misspecification, the presence of unmeasured or imprecisely measured confounders, and residual confounding likely explain at least part of the heterogeneity observed in the included studies. Potential for residual confounding also arises because of the long lag between exposure and disease and the potential consequences of subsequent pregnancies. Given the limitations of cross-sectional studies (e.g., temporal ambiguity, inclusion of prevalent cases), there also remains a need to examine the effect of gestational diabetes on the incidence of individual cardiovascular disease end points using longitudinal data. The composite end points used in the included studies were heterogeneous, which may explain some of the observed between-study variability, and some components may have been inappropriate (e.g., the inclusion of arrhythmias as an end point is debatable). Furthermore, it is possible that the observed associations were driven by one specific component of the composite end point.

The effect of gestational diabetes on the risk of incident cardiovascular disease independent of traditional cardiovascular risk factors (including type 2 diabetes) also warrants further investigation. Both Canadian cohort studies^12^,^13^ assessed the mediating effects of type 2 diabetes by including it as a covariate in their regression analyses, but this approach can result in biased estimates.^21^,^22^ Type 2 diabetes is both a confounder and a mediator of the relationship between gestational diabetes and cardiovascular disease, and the use of causal inference techniques such as marginal structural models^23^ is needed to address the potential mediation. Future studies should also examine the potential contribution of common genetic causes of gestational diabetes, type 2 diabetes, and cardiovascular disease.

To our knowledge, this is the first scoping review to examine the association between gestational diabetes and cardiovascular events. Previous systematic reviews have addressed the association between gestational diabetes and cardiovascular risk factors, such as hypertension, dyslipidemia, obesity, and blood pressure. 24–26 These reviews found that gestational diabetes had harmful effects on these particular cardiovascular risk factors. For example, Bentley-Lewis^15^ found that women with gestational diabetes had a higher risk of gestational hypertension than women without gestational diabetes (OR 1.34, 95% CI 0.49 to 3.71). In addition, Fraser et al.^4^ found that women with gestational diabetes had higher body mass index (34.66 v. 29.41 kg/m^2^, a difference of 5.25 kg/m^2^), waist circumference (97.95 v. 84.77 cm, a difference of 13.18 cm), systolic blood pressure (108.24 v. 103.12 mm Hg, a difference of 5.12 mm Hg), and fasting glucose (7.51 v. 4.93 mmol/L, a difference of 2.58 mmol/L) than women without gestational diabetes. Although these previous reviews did not address the effect of gestational diabetes on hard cardiovascular end points, their results are consistent with those of the present review.

Our study had a number of strengths. First, we followed a prespecified protocol in conducting the review and followed the guidelines set forth in PRISMA^8^ in reporting our results. Second, we contacted the authors of included studies to obtain additional information and to resolve important ambiguities regarding the included studies. Finally, we included conference abstracts (to minimize publication bias), as well as narrative reviews and editorials (to provide a complete summary of the existing literature examining this issue). In doing so, this comprehensive scoping review has identified key gaps in this literature that can be targeted in future research.

Our review also had some potential limitations. First, because of important heterogeneity in study design, overlap in study populations, and the limited data available, we were unable to perform a meta-analysis of data across studies. Second, for practical reasons, our search was restricted to studies published in English or French, which may have resulted in language bias. Finally, the study question is an etiologic one, and we were thus limited to the use of observational data. The possible effects of residual confounding must therefore be considered when these data are interpreted.

### Conclusion

Our scoping review of the available literature suggests that gestational diabetes mellitus is associated with an increased risk of cardiovascular disease. However, the available data are limited. Our examination of the role of subsequent diabetes in influencing this association indicates that the increased cardiovascular risk is likely mediated, in part, by an increased risk in subsequent diabetes, but this evidence remains inconclusive because of the sparse data.
